# 

**Published:** 1903-08-01

**Authors:** 


### NOTICE TO CORRESPONDENTS.

All MSS., Letters, Books for Review, and other matters intended for the Editor should be addressed The Editor, "The Hospital," The Hospital Buildings, 28 & 29 Southampton Street, Strand, W C.

The Editor cannot undertake to return rejected MS., even when accompanied by stamped directed envelope.

**Notice to Advertisers and Subscribers.**

All Advertisements, Orders for Copies of The Hospital, and Business Communications should be addressed to **THE MANAGER, (Not to the Editor)**, The Hospital, 28 & 29 Southampton Street, Strand, London, W.C.

The Cost of Subscription, post free, is as follows.—Per week, 3½d.; per quarter, 3s. 9d.; per half-year, 7s. 6d.; per annum, 15s. Foreign and Colonial:—Per annum, 19s. 6d., payable, in all cases, in advance.

**NOTICE.—Vol. XXXIII. of "The Hospital" (October 1902 to March 1903) in handsome cloth binding, will shortly be on sale at the office of this Journal, price 7s. 6d. Cases for binding the Numbers contained in this Volume 1s. 6d. Index to Vol. XXXIII., 2½d., post free.**

**The monthly part for August is now ready. Price 1s., by post 1s. 3d.**

### BOOKS RECEIVED.

H. J. Glaisher.

"Manual of Intragastric Technique." By J. Herschell, M.D.

"Polyphase Currents in Electrotherapy." By J. Herschell, M.D.

Longmans, Green and Co.

"Elementary Bacteriology." By M. L. Dhingra, M.D.

Whittaker and Co.

"The Apothecaries' Hall Manual." By M. Thomson.

Moorland's Press.

"Sparks. A Firebook for Nurses and Attendants." By R. C. Long.

J. Wright and Co.

"A Pharmacopœia for Diseases of the Skin." By James Startin.

Baillière, Tindall and Cox.

"Lessons on Massage." By Mrs. Palmer.

The Scientific Press, Limited.

"Sight and Hearing in Childhood." By R. Brudenell Carter and A. H. Cheatle.

### Scraps and Gleanings.

The town and district of Batley have presented to their ambulance brigade one of Wilson and Stockall's accident ambulances which won the silver medal at the Sanitary Congress at Bradford.

The Earl and Countess of Jersey entertained in July in the grounds of Osterley Park, 600 boys and girls and the staff from the various homes and ships of the National Refuges for Homeless and Destitute Children of which society Lord Jersey is President. The party, which was under the charge of Mr. H. Bristow Wallen, Secretary, included the 200 girls of the Sudbury and Ealing Homes; the boys of Fortescue House, Twickenham; boys from the Farm and Shaftesbury Schools, Bis'ey; and the sailor lads of the *Arethusa* and *Chichester* training ships. A special feature of the proceedings was the presentation by Lady Jersey of prizes given by her Royal Highness the Princess of Wales to those boys and girls who had best acted up to the motto of the Mayflower Guild, "Be good and do good."

### The Student of Medicine.

### APPOINTMENTS.

**W. Anderson,** M.B., C.M., Medical Officer of the Poplar Union Schools. **R. L. Bealy-Smith,** M.R.C.S., L.R.C.P.Lond., Certifying Factory Surgeon for the Bishopstoke District, Hants. **W. F. Victor Bonney,** M.S., M.D.Lond., Obstetric Registrar and Obstetric Tutor of the Middlesex Hospital. **William Brodie Brown,** M.B., C.M.Aberd., Medical Officer of Health for the Aboyne District of Aberdeenshire. **John S. Burton,** L.R.C.P., L.R.C.S.Edin., L.F.P.S.Glasg., Assistant Resident Medical Officer at the St. Mary's Hospital for Sick Children, Plaistow. **John Beattie Dunlop,** M.A.Cantab., M.R.C.S., L.R.C.P., Resident Medical Officer to the Royal United Hospital, Bath. **Charles Harold Dyer,** M.D. and C.M.Aberd., Medical Superintendent of the Checkheaton Small-pox Hospital, and reappointed Medical Officer of Health to the Checkheaton Urban District Council. **Jos. Wm. Gill,** M.R.C.S., L.R.C.P., D.P.H.Lond., Surgeon and Agent for the care of Sick and Wounded Seamen and Marines at Llantwit Major (Admiralty), and Medical Officer to the Nash Lighthouse Establishment by the Elder Brethren, Trinity House. **W. S. Handley,** M.S.Lond., F.R.C.S., Richard Hollins Cancer Research Scholar at Middlesex Hospital. **T. J. P. Hartigan,** F.R.C.S., Medical Officer in charge of the Light Department, X-Rays High Frequency, etc., at the Hospital for Diseases of the Skin, Blackfriars. **C. O. Hawthorne,** M.B., M.R.C.P., Examiner in Medicine and Clinical Medicine in the University of Aberdeen. **J. W. Holmes,** M.R.C.S., L.R.C.P., District Medical Officer of the Sheffield Union. **G. A. Johnston,** M.D.Brux., F.R.C.S.I., Certifying Factory Surgeon for the Ambleside District of Westmorland. **A. Campbell Keay,** M.B., Ch.B., Resident House-Surgeon to the Perth Infirmary. **James Kilbride,** L.R.C.P.I., M.R.C.S., Medical Referee under the Workmen's Compensation Acts for County Kildare. **W. S. Lazarus-Barlow,** M.D.Camb., F.R.C.P., Director of the Cancer Research Laboratories of the Middlesex Hospital. **A. E. Marwood,** L.R.C.P. and S.Edin., District Medical Officer of the Portsmouth Parish. **William McCallin,** M.D., R.U.I., D.P.H Camb., Assistant Medical Officer of Health to the Southampton Corporation. **H. L. Ormerod,** M.D., B.Ch., R.U.I., Certifying Factory Surgeon for the Westbury-upon-Trym District, Gloucester. **G. A. Peake,** L.R.C.P., L.S.A., M.R.C.S., L.D.S.Eng., Honorary Dental Surgeon to the Cheltenham General Hospital. **C. de C. Pellier,** M.A., B.C.Camb., District Medical Officer of the Malling Union. **W. Pemberthy,** M.R.C.S., L.R.C.P., District Medical Officer of the Wellington (Somerset) Union. **Frank Pershouse,** M.R.C.S., L.R.C.P.Lond., Certifying Factory Surgeon for the Stanford-le-Hope District, Essex. **C. H. Powers,** M.R.C.S., L.R.C.P., District Medical Officer of the Penrith Union. **T. Priestley,** M.R.C.S., L.R.C.P., District Medical Officer of the Sheffield Union. **T. Readman,** L.R.C.P. and S.Edin., District Medical Officer of the Lancaster Union. **John Robertson,** M.B.Edin., B.Sc., Medical Officer of Health for Birmingham. **W. F. Smeall,** M.B., Ch.B.Edin., Resident Medical Officer at the St. Mary's Hospital for Sick Children, Plaistow. **W. C. Spooner,** M.B., Ch.B.Edin., Certifying Factory Surgeon for the Blandford District, Dorset. **A. M. Watt,** M.B., C.M.Aberd., Medical Officer of the St. Olave's Union Children's Homes.

### "The Hospital" Institutional Library.

"Hospitals and Asylums of the World." 4 Vols., with a Portfolio of Plans, complete £12 12s.

"Burdett's Hospitals and Charities, 1902." 5s.

"Burdett's Official Nursing Directory, 1903." 3s. net.

"Cottage Hospitals, General, Fever, and Convalescent." 10s. 6d.

"Uniform System of Accounts for Hospitals and Public Institutions." New Edition. 4s. net.

"Hospital Expenditure: The Commissariat." 2s. 6d.

"A Legal Handbook for the Use of Hospital Authorities." 2s. 6d.

"The Cottage Hospital Case Book or Register of Patients." 10s. and 12s. 6d.

"The Furnishing and Appliances of a Cottage." 6d.

All these are published by the Scientific Press, Ltd., and may be obtained through any bookseller, or direct from the publishers, 28 and 29 Southampton Street, Strand, London, W.C.

Teale Fireplaces.—In our notice of the above in our issue of July 25th, Mr. R. Mousley Somers, junior partner in the Teale Fireplace Company, was, by mistake, referred to as Mr. R. Mousley.

### FROM A NURSE'S NOTE-BOOK.

Price 2/- post free.

The Diary and Experiences of a Hospital Nurse. The sketches in this little volume are vividly and dramatically written and reveal the powers of the authoress as a writer of fiction. For this reason the book must find interest with the general public as well as nurses. It is artistically bound.

### THE NURSE'S DICTIONARY OF MEDICAL TERMS AND NURSING TREATMENT.

Cloth 2/- post free; leather 2/6 net, 2/8 post free.

An indispensable companion for nurses.

### THE MIDWIVES' POCKET BOOK.

Price 1/- post free.

An admirable and useful little work recently revised.

### Other Works for Nurses.

### ELEMENTARY ANATOMY AND SURGERY FOR NURSES.

By McADAM ECCLES, F.R.C.S.

Price 2/6 post free.

Contains very clear and ample descriptions of these subjects.

### ELEMENTARY PHYSIOLOGY for NURSES.

By Dr. C. F. MARSHALL.

Price 2/- post free.

Dr. Marshall has succeeded in rendering his subject unusually interesting and easy of comprehension.

### NURSING: ITS THEORY AND PRACTICE.

By Dr. PERCY LEWIS.

Price 3/6 post free.

A very complete and lucid text-book.

*These books are obtainable of all Booksellers, and are published by* THE SCIENTIFIC PRESS, LIMITED, 28 & 29 Southampton Street, Strand, London, *who will send their latest Catalogue of Nursing Text-books post free to any Nurse on application.*

